# *Moringa oleifera* Leaf Powder as New Source of Protein-Based Feedstuff Improves Growth Performance and Cecal Microbial Diversity of Broiler Chicken

**DOI:** 10.3390/ani13061104

**Published:** 2023-03-20

**Authors:** Haiwen Zhang, Liangmin Huang, Shihui Hu, Xinyun Qin, Xuemei Wang

**Affiliations:** 1 Key Laboratory of Tropical Animal Breeding and Epidemic Disease Research of Hainan Province, Hainan University, Haikou 570228, China; hwzhang@hainanu.edu.cn (H.Z.);; 2 Laboratory of Tropical Animal Breeding, Reproduction and Nutrition, Hainan University, Haikou 570228, China

**Keywords:** *Moringa oleifera* leaf powder, broiler chickens, cecal microbiota, growth performance, Bacteroidetes, Firmicutes

## Abstract

**Simple Summary:**

In this study, we investigated the effects of *Moringa oleifera* leaf powder (MOLP) on growth performance, carcass characteristics, and cecum micro-organisms of broiler chickens by using different levels of MOLP instead of canola cake. The results showed that 5% MOLP had an improving effect on broiler growth performance and carcass characteristics in the early and late stages. The sequence of cecal microbiota from broiler chickens via 16S rRNA revealed that 5% MOLP is likely to enhance the growth performance and carcass characteristics of broiler chickens by regulating the relative abundance of intestinal flora. The results of this study can provide some reference for the application of MOLP in livestock and poultry farming.

**Abstract:**

Currently, the lack of protein source feed has become a pressing issue. *Moringa oleifera* leaf powder (MOLP) has good potential for the development of protein-derived feeds due to its good protein quality and abundance, but little is known about its effects on broiler growth performance and cecal microbiota. In this study, the chickens were fed different rates of MOLP (1%, 3%, 5%, 7%, and 9%) instead of the rape seed cake, and the effects of different levels of MOLP on growth performance, carcass characteristics, and cecal microbiota of the broilers were evaluated at two different growth stages (day 28 and day 56). In terms of growth performance, the best results were obtained at the 3% MOLP level in the early stages (*p* < 0.05). In terms of carcass characteristics, in the early stage, the level of 5% MOLP had the best effect; in the later stage, 5% MOLP also had the best effect. In terms of cecal microbial changes, the alpha diversity analysis revealed that 5% MOLP enhanced the richness and diversity of broiler intestinal flora. At the phylum level, the addition of 5% MOLP adjusted the relative abundance of Firmicutes and Bacteroidetes to a level close to that of the A1 group on day 28, while 5% MOLP significantly reduced the relative abundance of Bacteroidetes (*p* < 0.05) compared to the A2 group on day 56, and the relative abundance of Firmicutes was still higher in the D2 group than in the A2 group (*p* < 0.05). At the genus level, MOLP addition consistently and significantly increased the relative abundance of Bacteroides (*p* < 0.05), except for 3% on day 28 and 1% on day 56. For Oscillospira, increasing MOLP levels in the pre- and post-period resulted in a significant decrease in the relative abundance of Oscillospira (*p* < 0.05). In conclusion, MOLP helps to enhance growth performance and carcass characteristics and improve the cecal microbial structure of broilers. The recommended rate of MOLP addition for broilers is 5% in both the early and late stages.

## 1. Introduction

*Moringa oleifera* (Capparales: Moringaceae), also called drumstick tree, originated in India, Africa, and Southeast Asia [1]. *Moringa oleifera* is considered an important food plant of high nutritional value, and almost all parts are edible [2]. The leaves of *M. oleifera* have active constituents with multiple bioactivities; quercetin is the predominant flavonol in the leaves of *M. oleifera* and exhibits multiple therapeutic properties as a potent antioxidant [3]. Chlorogenic acid is another important active compound in *Moringa* and could promote glucose metabolism in rats [4]. *Moringa oleifera* leaves are an extremely valuable food source for both humans [5] and animals [6,7]; they are reported to be rich in highly digestible protein, vitamins, and essential amino acids [8], and dried leaves have crude protein up to 30.3% and 19 amino acids [9]. Additional advantages of *M. oleifera* are that it is resistant to drought, fast growing, and easy to cultivate in tropical areas, and it may serve as an alternative source of food [3]. *Moringa* leaves also exhibit antimicrobial properties that inhibit bacterial growth [10], and ethanol extracts have shown broad spectrum activity against pathogens such as *Escherichia coli*, *Pseudomonas aeruginosa*, *Staphylococcus aureus*, and *Enterobacter aerogenes* [11].

To evaluate the potential of *Moringa oleifera* leaf powder as a feed, the effects of *Moringa oleifera* leaf powder (MOLP) have been investigated in some animals. Although MOLP supplemented in a 20% ratio in the diet did not influence rat growth performance [12], MOLP successfully replaced commercial components in concentrate feeds as a protein source for dairy cows [13], and MOLP substituted for alfalfa meal at 20% significantly improved rabbit growth performance, meat quality, and antioxidant and biochemical parameters [14]. Additionally, a corn-based diet supplemented with 1.2% MOLP significantly improved broiler chicken growth performance, intestinal microarchitecture, and acidic mucin production [15], and a study of the effects of MOLP in a ratio of 10% MOLP in 3 strains of chickens showed that MOLP addition positively affected growth performance and carcass characteristics [16]. However, to our knowledge, there are few reports on the effects of MOLP on chicken cecal microflora.

The cecum is the main site of intestinal fermentation in chickens, has the highest abundance of microorganism species, and is one of the most important factors that affect animal health and growth performance. Therefore, it is necessary to study the cecal microbial diversity of chickens [17]. Sequencing of 16S rRNA has been widely used to study microbial diversity and can be used to maximize bacterial classification [18]. Early culture-independent methods and denaturing gradient gel electrophoresis (DGGE)-based techniques were initially applied to analyze intestinal microbiota, but they were time-consuming and had limited coverage. However, with the development of sequencing technology, next-generation sequencing (NGS) could offer unparalleled coverage and depth at low a cost [19].

As a valuable potential feed resource, little is known about the influence of MOLP and suitable substitution levels for the cecal microbiota of chicken. Therefore, in this study, different levels of MOLP substitution for rapeseed cake were fed to broiler chickens. First, the effects of different levels of MOLP substitution on growth performance and carcass characteristics of broiler chickens at 2 growth stages (day 28 and day 56) were evaluated. Second, the effects of different levels of MOLP substitution on the cecal microbiota of broiler chickens at two growth stages were evaluated by using the NGS technique.

## 2. Materials and Methods

### 2.1. Experimental Design

All procedures of chicken raising and cecum content sampling, evaluation of broiler growth, and carcass characteristics were approved by the animal care and use committee of Hainan University. During the execution and sampling process, we exerted all efforts to minimize the suffering of the animals. A total of 216 male broiler chickens with similar weight (2 weeks) were randomly divided into 6 groups, each group had 3 repetitions, and each repetition had 12 individuals. Chickens were fed for 28 and 56 days with different feed formulas. As listed in Table 1, the A1/A2 group was fed the basic diet, and the B1/B2, C1/C2, D1/D2, E1/E2, and F1/F2 groups were fed an experimental diet substituted with 1%, 3%, 5%, 7%, and 9% MOLP for an equal ratio of rape seed cake. The details of the experiment are as follows: during the experimental period, the routine procedure was sterilized, the ambient temperature was gradually decreased from 34 °C to a constant 26 °C, natural light was provided, feed was added once a day at 8:00 am and once a day at 15:00 pm, and the broilers were free to feed and drink during the period. To keep the consistency of energy and protein levels, several ratios of feedstuff were slightly adjusted, such as maize and soybean meal.

### 2.2. Measurement of Growth Performance

After fasting 12 h in advance on day 28 and day 56 of the test period, respectively, 3 broilers in each group were weighed by replicates, the total body gain (TBG) of each group was counted, and the feed intake of each group was weighed at the same time to calculate the feed/gain (F/G) of broilers in each group at different stages for comparative analysis. The average daily gain (ADG), average daily feed intake (ADFI), and F/G were calculated with the following formulas.

ADFI = total food intake/(trial days × number of test animals)

ADG = (final weight − initial weight)/measured days

F/G = total feed consumption/(final weight − initial weight)

### 2.3. Measurement of Carcass Characteristics

Eighteen test broilers were slaughtered on day 28 and day 56, respectively, and the slaughter rate, half-cleaning rate, and full-cleaning rate of broilers were subsequently measured according to the methods in “Poultry Production Performance Nomenclature and Metric Statistical Methods (NY/T823-2004)”.

### 2.4. Cecum Sample Collection

The sampling period was divided into 2 stages: 28 days and 56 days. All groups were starved for 12 h before sampling, and 1 chicken from each repetition (with body weight closest to the mean weight) was selected and sacrificed. Samples were aseptically scraped from cecum mucosa and placed in the sterile tube, and all samples were immediately stored at −80 °C for further analysis.

### 2.5. DNA Extraction and 16S rRNA Gene Sequencing

Total genomic DNA was extracted from cecal samples by using the stool DNA Kit (Sigma-Aldrich, Taufkirchen, Germany) according to the manufacturer’s instructions. The V4-V5 regions of bacterial 16S rRNA gene (from 507 to 907) were amplified from extracted DNA by using barcoded primers 515F (5′-GTGCCAGCMGCCGCGG-3′) and 907R (5′-CCGTCAATTCMTTTRAGTTT-3′). The PCR reaction was carried out in a 50 μL system with 10 μL 1× primeSTAR buffer (Mg^2+^ Plus), 4 μL 200 μM dNTP mixture, 1 μL 0.1 μM forward and reverse primers, 0.5 μL 1.25U primer STAR HS DNA polymerase, 10 ng template DNA, and ddH_2_O to the final volume of 50 μL. The PCR reaction was carried out by using PCR amplification (GeneAmp PCR System 9700, Foster City, CA, USA). The PCR reaction parameters were as follows: following the denaturation stage at 98 °C for 1 min, the amplifications were carried out with 27 cycles at the melting temperature of 98 °C for 30 s, an annealing temperature of 55 °C for 30 s, and an extension temperature of 72 °C for 30 s. Furthermore, an extra extension step was performed at 72 °C for 5 min. The amplicons were pooled and quantified by using Nanodrop (Thermo Scientific, Carlsbad, CA, USA). Then, the DNA library was sequenced by using the PCR-free method, and next-generation sequencing was performed via an Illumina Hiseq PE250 (SAGENE, Guangzhou, China).

### 2.6. Quality Control

The barcodes were cut off, and pair-end tags (reads 1 and reads 2) of each sample were joined by using the Pandaseq program. Raw tags of continuously low-quality value (quality threshold ≤ 19) and the base number reached the set length (length value set as 3). Then, the first low quality base site was truncated, the cut-out tags data set that has continuously high-quality bases with length <75% of the tag length was filtered. The pair-end tags without overlap were filtered, and the filtered tags were homogenized; finally, the possible chimeras were recognized and filtered.

### 2.7. Bioinformatics and Statistical Analysis

Broiler cecum samples were collected as a mixed sample of 3 birds per group, and 3 replicates were performed in each group. The high-throughput sequences were clustered into operational taxonomic units (OTU) with similarity of at least ≥97%. The distribution of OTU tags in each sample was analyzed by using a box plot, and the heat map of abundance of clustering and principal component analysis (PCA) was plotted based on the table of OTU abundance. Cladogram analysis was performed to blast representative OTU tags through Figure Tree software. The α-diversity of community richness (including ACE and Chao1) and diversity (including Shannon and Simpson) was ascertained by using MOTHUR [20]. The taxonomic assignments of the OTUs were performed by using QIIME software (quantitative insights into microbial ecology) based on the databases of SILVA [21], Greengene [22], and RDP [23]. The microbial significance analysis between groups was performed by using the effect size (LEfSe) method and linear discriminant analysis (LDA). Statistical analysis was performed by using SPSS 18.0 *t*-tests (SPSS Inc., Chicago, IL, USA), and *p* < 0.05 was considered significant.

## 3. Results

### 3.1. Effect of Different MOLP Levels on the Growth Performance of Broiler Chickens

On 28 days, as shown in Table 2, the daily weight gain of the B1, C1, and D1 groups increased significantly. Furthermore, there was a significant difference compared to the E1 and F1 groups (*p* < 0.05), but there was no significant difference between the B1, C1, and D1 groups (*p* > 0.05). Group C1 had the highest daily food intake value and was significantly different from the other groups (*p* < 0.05), while group A1 had the lowest value of 59.91 g. The daily feed intake of group A1 was significantly different from all other groups (*p* < 0.05), except that there were no significant differences with group D1 (*p* > 0.05). In terms of total weight gain, the B1 and C1 groups had higher values of 13.56 kg and 13.52 kg, respectively. There were no significant differences between the above two groups (*p* > 0.05), but there was a significant difference with the D1, E1, and F1 groups (*p* < 0.05). In the material-to-weight ratio, the values were lower in group B1 and C1, and there was no significant difference between the above two groups (*p* > 0.05), but there was a significant difference with the D1, E1, and F1 groups (*p* < 0.05). On day 56, as shown in Table 2, there were no significant differences in daily weight gain, daily feed intake, total weight gain, and feed-to-weight ratio between the groups (*p* > 0.05).

### 3.2. Effect of Different MOLP Levels on Broiler Chicken Carcass Characteristics

On day 28, as shown in Table 3, the different levels of MOLP did not have a significant effect (*p* > 0.05) on the preslaughter weight, the half-clean bore weight, the slaughter rate, or the full-clean bore weight of broilers compared to the control group. Significant differences (*p* < 0.05) existed in the slaughter weight and semi-clear bore weight in groups C1 and D1 compared to group E1, while no significant differences (*p* > 0.05) existed in both of these indicators in the other groups. The slaughter rate in group E1 was significantly different from that in group A1, C1, D1, and F1 (*p* < 0.05), while the rest of the groups were not significantly different from each other (*p* > 0.05). There was a significant difference (*p* < 0.05) between group E1 and all other groups in the semi-clean chamber rate, except that there was no significant difference (*p* > 0.05) between group E1 and group B1 in the semi-clean chamber rate. Although there was a significant difference (*p* < 0.05) between groups C1 and D1 compared to groups B1 and E1, there was no significant difference between the C1 and D1 groups, but the semi-net bore rate was higher in the C1 group. On day 56, as shown in Table 4, different levels of MOLP did not have a significant effect on the slaughter rate and total net bore weight of the broilers compared to the control group (*p* > 0.05), and with increasing MOLP levels, the values of pre-slaughter weight, slaughter rate, half net bore weight, and total net bore weight of the D2 group were the highest among all groups, and there were significant differences between them and the A2 and B2 groups (*p* < 0.05). In the semi-net bore rate, there was a significant difference only between the E2 and C2 groups (*p* < 0.05), and the value was higher in the E2 group, while there was no significant difference between the rest of the groups (*p* > 0.05). 

### 3.3. Qualified Sequence Tags

**Figure 1:**
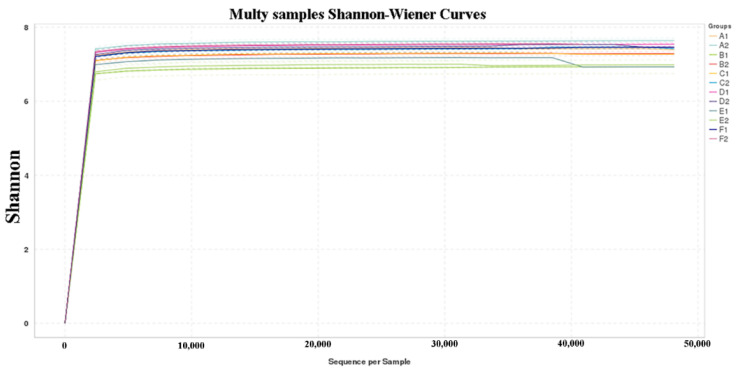
Shannon value and rarefaction curves of OTUs clustered at 97% sequence identity across different samples. A1–F1 represented the addition level of 1–9%, respectively, at day 28; A2–F2 represented the addition level of 1–9%, respectively, at day 56, the same as below.

The sequencing results were divided into two stages: day 28 and day 56. The total qualified sequences of the cecal samples of 18 broiler chickens of each stage were 292,319 and 276,672, and there was an average of 48,719 and 46,112 reads per cecal sample, respectively. The total sequences corresponded to 6554 and 6576 OTUs, with an average of 1092 and 1096 OTUs per sample, respectively. The Shannon index (Table 5) and rarefaction curves (Figure 1) for each sample reached the saturation plateau, which showed that our sampling had sufficient sequence coverage to accurately describe the bacterial composition of each group. The indices of bacterial richness (Chao and Ace) and bacterial diversity (Shannon and Simpson) are shown in Table 5.

### 3.4. Taxonomic Composition

**Figure 2:**
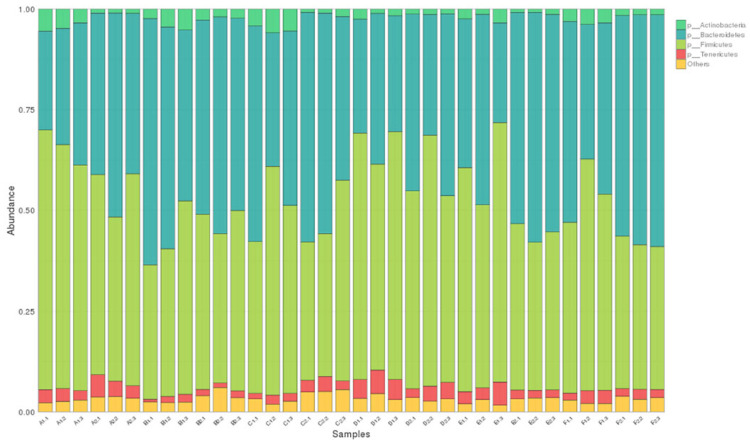
Distribution of the taxonomy stack of the microflora in the cecum of broiler chickens at the phylum level. The colored bar graph shows the distribution of the level of the bacterial phylum in different treatment groups, and 1-1 to 1-3 represented the repetitions of the A1 group, which is the same as for other groups.

**Figure 3:**
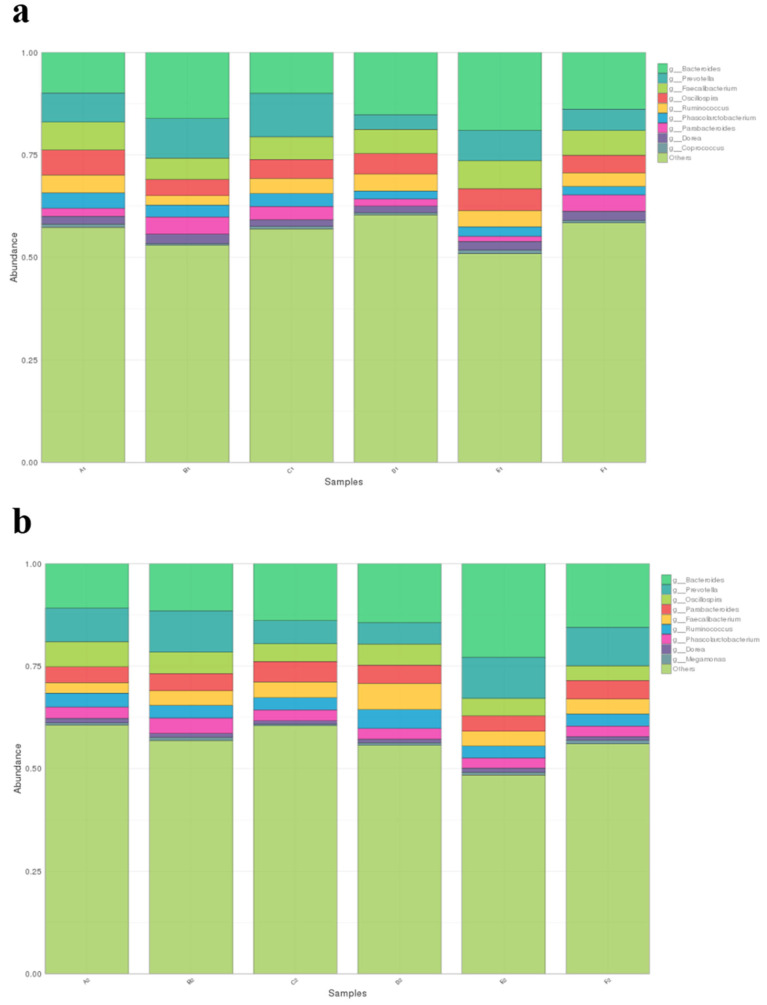
(**a**,**b**)Distribution of the taxonomic stack of the microflora in the cecum of broiler chickens at the genus level. The colored bar graph shows the distribution of the level of the bacterial genus in different treatment groups.

All filtered tags were classified from phylum to species based on the SILVA taxonomic database and by using QIIME. Bacteria with a relative abundance ≥1% were considered dominant, as shown in Table 6 and Table 7, and were identified in two different stages of broiler chicken growth. The dominant microflora at the phylum level were Firmicutes, Bacteroidetes, Actinobacteria, and Tenericutes (Figure 2), and at the genus level, they were *Ruminococcus*, *Bacteroides*, *Dorea*, *Faecalibacterium*, *Oscillospira*, *Parabacteroides*, *Phascolarctobacterium*, *Prevotella*, *Coprococcus*, and *Megamonas* (Figure 3). Because only one kind of bacteria was identified at the kingdom level and two kinds of bacteria were identified at the species level, we focused mainly on the phylum and genus level; therefore, the kingdom and species levels were not listed in Table 6 and Table 7.

### 3.5. Effect of Different MOLP Levels on Microbial Community: The Relative Abundance and Diversity of the Microbial Community at the Phylum Level

**Figure 4:**
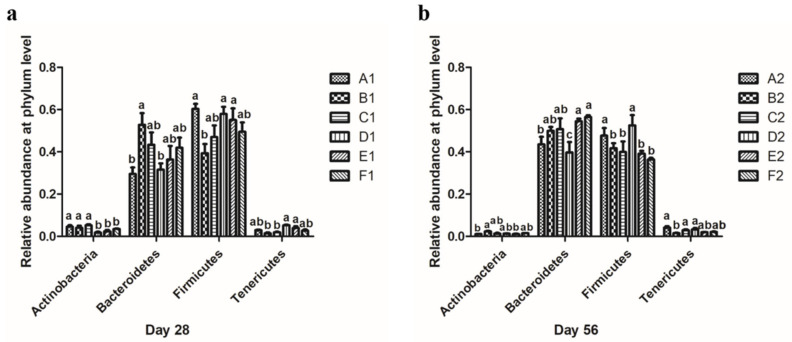
Effects of different additions of MOLP on the relative abundance of (**a**) the most dominant phylum (≥1%) on day 28 and (**b**) the most dominant phylum (≥1%) on day 56 in the cecum microflora of broiler chickens. The error bars represent the SEM of three samples, and the results are described as mean ± SEM. Statistical analysis of the data was performed by using Duncan’s method to analyze the significance of differences between samples by using IBM SPSS software. Means with similar superscripts in the same column indicate that there are no significant differences (*p* > 0.05), and those with different superscripts in the same column indicate significant differences (*p* < 0.05).

In this study, a relative abundance of >1% was considered dominant, and the effect of MOLP on microbial communities was evaluated at the phylum and genus levels. As shown in Figure 4a, the proportions of Actinobacteria in the D1 (0.018 ± 0.004), E1 (0.024 ± 0.006), and F1 (0.035 ± 0.002) groups were significantly lower than those of the A1 (0.046 ± 0.006), B1 (0.041 ± 0.009), and C1 (0.052 ± 0.005) groups (*p* < 0.05). The proportion of Bacteroidetes in the B1 group (0.528 ± 0.055) was significantly higher than that of the A1 (0.295 ± 0.031) and D1 (0.315 ± 0.030) groups (*p* < 0.05), whereas C1 (0.433 ± 0.059), E1 (0.363 ± 0.065), and F1 (0.419 ± 0.048) groups did not differ significantly from the A1, D1, and B1 groups. The proportions of Firmicutes in the A1 (0.603 ± 0.024), D1 (0.579 ± 0.034), and E1 (0.551 ± 0.055) groups were significantly higher than those of the B1 (0.393 ± 0.044) group (*p* < 0.05), whereas there was no significant difference between the C1 (0.470 ± 0.055) and F1 (0.495 ± 0.044) groups and the A1, B1, D1, and E1 groups. On day 56 (Figure 4b), the proportion of Actinobacteria in B2 (0.024 ± 0.002) was significantly higher than in the A2 (0.011 ± 0.001) and E2 (0.011 ± 0.002) groups (*p* < 0.05), and no significant differences were found between the C2 (0.013 ± 0.003), D2 (0.013 ± 0.001), and F2 (0.015 ± 0.001) groups and the A2, B2, and E2 groups. The proportions of Bacteroidetes in the E2 (0.544 ± 0.013) and F2 (0.564 ± 0.009) groups were significantly higher than those of the A2 (0.435 ± 0.036) and D2 (0.397 ± 0.049) groups (*p* < 0.05). The D2 and A2 groups were also significantly different from each other (*p* < 0.05), but there were no significant differences between the B2 (0.499 ± 0.019) and C2 (0.507 ± 0.051) groups and the A2, E2, and F2 groups. The proportions of Firmicutes in the A2 (0.476 ± 0.036) and D2 (0.525 ± 0.049) groups were significantly higher than in the B2 (0.417 ± 0.024), C2 (0.399 ± 0.050), E2 (0.391 ± 0.01), and F2 (0.364 ± 0.007) groups (*p* < 0.05).

### 3.6. Effect of Different MOLP Levels on the Relative Abundance and Diversity of the Microbial Community at the Genus Level

**Figure 5:**
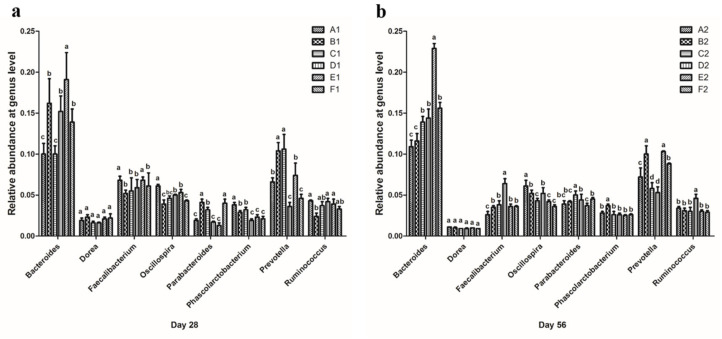
Effects of different additions of MOLP on relative abundance of (**a**) the most dominant genus (≥1%) on day 28 and (**b**) the most dominant genus (≥1%) on day 56 in the cecum microflora of broiler chickens. The error bars represent the SEM of three samples, and the results are described as mean ± SEM. Statistical analysis of the data was performed by using Duncan’s method to analyze the significance of differences between samples by using IBM SPSS software. Means with similar superscripts in the same column indicate that there are no significant differences (*p* > 0.05), and those with different superscripts in the same column indicate significant differences (*p* < 0.05).

At the genus level, as shown in Figure 5a, the proportion of *Bacteroides* in the E1 group (0.191 ± 0.033) was significantly higher than in other groups, and the B1 (0.162 ± 0.030), D1 (0.152 ± 0.019), and F1 (0.139 ± 0.016) groups had significantly higher proportions than the A1 (0.100 ± 0.013) and C1 (0.100 ± 0.010) groups. The proportions of *Faecalibacterium* in the A1 (0.068 ± 0.005) and E1 (0.068 ± 0.004) groups were significantly higher than those in the B1 (0.052 ± 0.004), C1 (0.055 ± 0.016), D1 (0.059 ± 0.010), and F1 (0.061 ± 0.016) groups (*p* < 0.05). The proportion of *Oscillospira* in the A1 group (0.061 ± 0.007) was significantly higher than in the other groups (*p* < 0.05), and the D1 (0.050 ± 0.001) and E1 (0.053 ± 0.004) groups had significantly higher proportions than the B1 (0.039 ± 0.005) and F1 (0.043 ± 0.001) groups (*p* < 0.05). The proportion of *Parabacteroides* in the B1 (0.041 ± 0.004) and F1 (0.040 ± 0.005) groups was significantly higher than in other groups (*p* < 0.05), and the proportion of *Parabacteroides* of the C1 (0.032 ± 0.003) group was significantly higher than in the A1 (0.019 ± 0.002), D1 (0.017 ± 0.001), and E1 (0.013 ± 0.003) groups (*p* < 0.05). The proportion of *Phascolarctobacterium* in the A1 (0.038 ± 0.003) group was significantly higher than in the other groups (*p* < 0.05), and the B1 groups (0.029 ± 0.002) and C1 (0.032 ± 0.003) groups had significantly higher proportions than the groups D1 (0.019 ± 0.002), E1 (0.023 ± 0.003), and F1 (0.021 ± 0.003) (*p* < 0.05). The proportions of *Prevotella* in the B1 (0.104 ± 0.010) and C1 (0.106 ± 0.018) groups were significantly higher than those of the other groups (*p* < 0.05), and the A1 (0.066 ± 0.005) and E1 (0.074 ± 0.015) groups had significantly higher proportions than did the D1 (0.036 ± 0.005) and F1 (0.046 ± 0.005) groups (*p* < 0.05). Finally, the proportion of *Ruminococcus* in the A1 (0.043 ± 0.001), D1 (0.042 ± 0.004), and E1 (0.039 ± 0.006) groups was significantly higher than that of the B1 group (0.024 ± 0.004) (*p* < 0.05), whereas no significant differences were found between the C1 (0.037 ± 0.005) and F1 (0.033 ± 0.003) groups and the other four groups.

On day 56 (Figure 5b), the percentage of *Bacteroides* in the E2 group (0.229 ± 0.006) was significantly higher than in other groups (*p* < 0.05), and the C2 (0.139 ± 0.007), D2 (0.144 ± 0.011), and F2 (0.156 ± 0.007) groups had significantly higher proportions than the A2 (0.109 ± 0.008) and B2 (0.116 ± 0.009) groups (*p* < 0.05). The proportion of *Faecalibacterium* in the D2 group (0.064 ± 0.006) was significantly higher than that of other groups (*p* < 0.05), and the proportion in the A2 group (0.026 ± 0.004) was significantly lower than in the B2 (0.035 ± 0.002), C2 (0.038 ± 0.005), E2 (0.036 ± 0.003), and F2 (0.036 ± 0.001) groups (*p* < 0.05). The proportion of *Oscillospira* in the A2 group (0.061 ± 0.007) was significantly higher than in other groups (*p* < 0.05), whereas the B2 (0.052 ± 0.004) and D2 (0.052 ± 0.007) groups had significantly higher proportions than the C2 (0.043 ± 0.003), E2 (0.042 ± 0.002), and F2 (0.036 ± 0.002) groups (*p* < 0.05). The proportion of *Parabacteroides* in the C2 group (0.050 ± 0.005) was significantly higher than in the other groups (*p* < 0.05), and there were significant differences between the D2 (0.044 ± 0.007), F2 (0.045 ± 0.002), and E2 (0.037 ± 0.003) groups (*p* < 0.05), but the A2 (0.039 ± 0.004) and B2 (0.042 ± 0.001) groups were not significantly different from the D2, E2, and F2 groups. The proportion of *Phascolarctobacterium* in the B2 group (0.037 ± 0.002) was significantly higher than in other groups (*p* < 0.05). *Prevotella* proportions in the B2 (0.100 ± 0.010) and E2 (0.103 ± 0.001) groups were significantly higher than those in the A2 (0.072 ± 0.011), C2 (0.058 ± 0.007), D2 (0.053 ± 0.007), and F2 (0.088 ± 0.001) groups (*p* < 0.05). The proportion of the F2 group was significantly higher than that of the A2, C2, and D2 groups (*p* < 0.05); and the proportion of the A2 group was significantly higher than that of the C2 and D2 groups (*p* < 0.05). Finally, the proportion of *Ruminococcus* in the D2 group (0.046 ± 0.005) was significantly higher than in the other five groups (*p* < 0.05).

### 3.7. Effect of Different MOLP Levels on the Clustering of Cecal Microflora at the Genus Level

**Figure 6:**
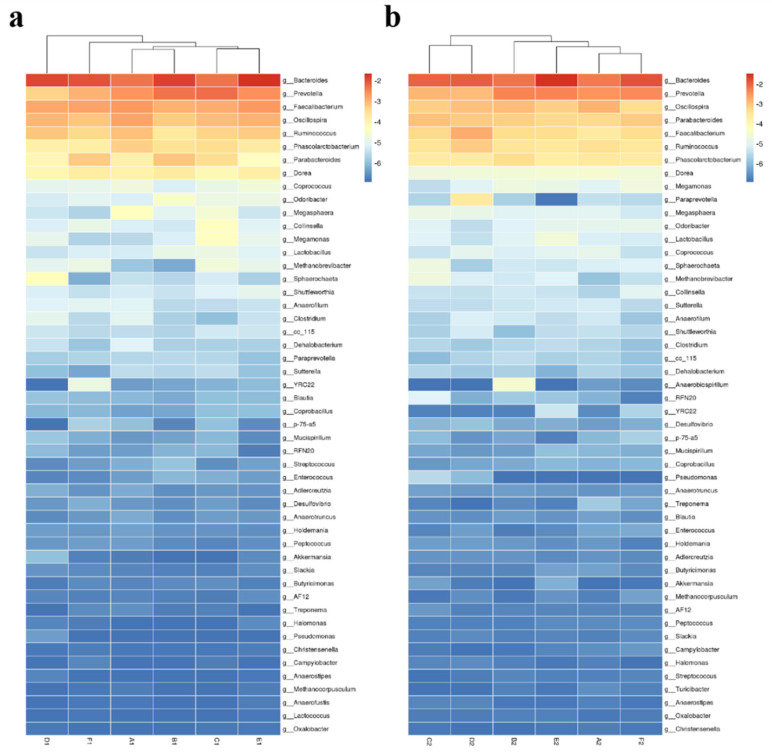
The heat map (grouped by column, the bacteria were screened and ranked as the top 50 of the relative abundance) of the microbial composition in the cecum of broiler chickens at the genus level of (**a**) day 28 and (**b**) day 56. The heat map indicates the relative abundance of each genus in different groups, and the darkness of the color indicates the ranking: the darkest red marks the highest value, and the darkest blue marks the lowest value.

The heat map shown in Figure 6 shows the different cecal microbial groups clustered at the genus level by column. At the early stage (Figure 6a), the A1 group had a similar microbial proportion to that of the B1 group, and the D1 group had a significantly different microbial proportion compared to the A1 group, indicating that a 5% increase in the cecal microbiota induced the most significant change during the early stage. However, in the later stage (Figure 6b), the A2 group had a similar microbial proportion to the F2 group, and the C2 group had a significantly different microbial proportion compared to the A2 group, which showed that an addition level of 3% caused the most significant change in the cecal microbiota, while a higher addition level resulted in a cecal microflora proportion similar to that of the A2 group.

### 3.8. Effect of Different MOLP Levels on the Predominant Taxa of Each Group

**Figure 7:**
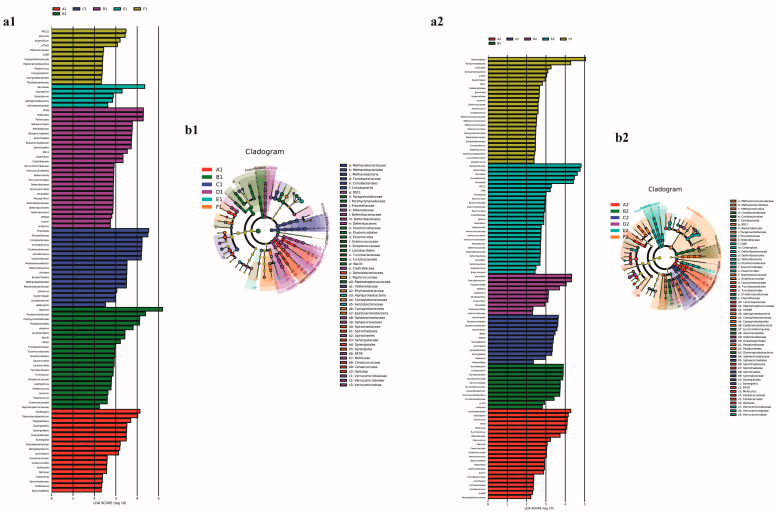
LEfSe identified the most differentially abundant taxa of each group. (**a1**) The LDA score at day 28, (**b1**) the cladogram at day 28, (**a2**) the LDA score at day 56, and (**b2**) the cladogram at day 56. The taxonomic cladogram obtained from LEfSe analysis of 16S sequences (relative abundance ≥0.1%). The length of the histogram represented the effect size of different bacteria and the higher score of LDA of the bacteria represents the more dominant bacteria in the sample. The circle from inside to outside represented the taxonomic rank from kingdom to species in the cladogram; the size of the circle was positively correlated to the abundance. Only taxa that meet an LDA score > 2 and *p* < 0.05 are shown.

As shown in Figure 7, linear discriminant analysis effect size analysis (LEfSe) was used to identify the predominant taxa of each group. In the early stage (Figure 7a1), tested at different taxon levels, the most dominant bacteria in the A1 to F1 groups were *Oscillospira*, Paraprevotellaceae, *Prevotella*, RF39, Barnesiae, and YRC22, respectively. Alternatively, the most dominant bacteria during the later stage (Figure 7a2) in the A2 to F2 groups were Lachnospiraceae, Actinobacteria, Spirochaetes, Faecalibacterium prausnitzii, Bacteroidaceae, and Bacteroidetes, respectively. All dominant bacteria in each group could be distinguished by biomarkers.

## 4. Discussion

The composition of the intestinal microbiota of chickens is directly related to growth performance and health [24,25]. As a potential superior feed resource, the substitution of rapeseed cake with different MOLP levels can have numerous advantages, and suitable replacement levels must be confirmed by analyzing growth performance, carcass characteristics, and changes in the cecal microbiota at different growth stages (day 28 and day 56).

In terms of growth performance, an MOLP level of 1–5% in the early stage had a certain enhancement effect on daily weight gain, daily feed intake, and total weight gain of broilers, although in the later stage, there were no significant difference (*p* > 0.05) in all indicators of growth performance compared to the control group, but the growth effect of broilers in the test and control groups was similar. In terms of carcass characteristics, the slaughter rate of broilers reared at different levels of MOLP levels in the early and late stages was 83.6%, and the whole-clean-bore rate was above 62.5%. Both of these results showed that the use of MOLP as a protein ingredient for broiler rearing is feasible.

In terms of changes in the cecal microbiota, as shown in Figure 1, the Shannon index and rarefaction curves for each sample reached the saturation plateau, which indicated that the sequencing depth of each sample had sufficient sequence coverage to accurately describe the bacterial composition. Usually, the number and structural composition of the organism’s intestinal flora have an important impact on the digestion, absorption, and health of the host. In the analysis of intestinal flora, the Ace and Chao indices are used to reflect the richness of the intestinal flora, and their values are proportional to the richness of the intestinal flora. The Shannon index and the Simpson index are related to the diversity of intestinal flora. The larger the Shannon index and the smaller the Simpson index, the higher the diversity of intestinal flora, and the richness and diversity of intestinal flora are positively correlated with the stability of the flora and the ability to resist pathogenic infection [26]. In this experiment, only the average values of the Chao, Ace, and Shannon index of the D1 and F1 groups were higher than those of the A1 group in the early stage, while the Simpson index values were equal to those of the A1 group, but the average values of the Chao, Ace, and Shannon indexes of D1 were higher than those of the F1 group, indicating that the addition of 5% MOLP in the early stage could better enhance the richness and diversity of the intestinal flora of the broiler. The results indicated that the addition of 5% MOLP in the first stage could better enhance the richness and diversity of the broiler intestinal flora. Although the addition of 9% MOLP had similar effects on the richness and diversity of the intestinal flora of broiler chickens, their performance in terms of growth performance and carcass characteristics was not significantly different from that of the A1 group, while the performance of the D1 group in terms of growth performance and carcass characteristics improved significantly compared to that of the A1 group. The reason may be that the digestive system of broilers in the early stage has not been perfected, so there is a certain delay in adapting to the high level of MOLP, although the abundance and diversity of the intestinal flora were increased to some extent, but it takes some time for broilers to transform the nutrients into their own metabolites. Therefore, the performance of the F1 group was not better than that of the A1 group in terms of growth and carcass characteristics, although the abundance and diversity of the flora were higher than those of the A1 group. In the later period, only the mean Chao and Ace indices of the D2 group were higher than those of the A2 group, while the mean Shannon index of the D2 group was lower than that of the A2 group, and the mean Simpson index was equal to that of the A2 group. The mean Chao, Ace, and Shannon indices of the other groups were lower than those of the A2 group, while the mean Simpson index was equal to or close to that of the A2 group. Given that at the later stage, although the D2 group did not differ significantly from the A2 group in growth performance, the D2 group performed better in carcass characteristics, the optimal rate of MOLP addition at the later stage was still 5%.

At the phylum level, the composition of the intestinal flora consisted mainly of Firmicutes and Bacteroidetes, and in this study, the dominant phylum of fecal micro-organisms was the same in all test groups, and the most abundant phylum was Firmicutes, Bacteroidetes, Actinobacteria, and Tenericutes; these findings are consistent with those of a previous report [27,28]. Current studies have shown that an increase in the abundance of Firmicutes has various effects, such as promoting fiber decomposition by intestinal flora, promoting energy absorption from food, and improving host body weight; while an increased abundance of Bacteroidetes may be related to host lean body size [19,29]. The results of this study showed that the addition of different levels of MOLP had inconsistent effects on the relative abundance of Firmicutes. On day 28, the addition of 1% MOLP significantly reduced the relative abundance of Firmicutes (*p* < 0.05), and higher MOLP levels resulted in a higher relative abundance of Firmicutes. However, the relative abundance of Firmicutes was lower in all test groups than in the control group, while the relative abundance of Firmicutes in the D1 group was closest to that of the A1 group. The relative abundance of Bacteroidetes in the test group was numerically higher than that of the control group, and interestingly, the relative abundance of Bacteroidetes in the D1 group was also the closest to that in the A1 group. Furthermore, we observed that the D1 group had the lowest relative abundance of Actinobacteria among all groups and the highest relative abundance value of Tenericutes among all groups in the data at day 28. On day 56, the relative abundance of Bacteroidetes was higher in all test groups than in the A2 group, except for 5% MOLP, which significantly reduced the relative abundance of Bacteroidetes (*p* < 0.05), while the relative abundance of Firmicutes was higher only in the D2 group than in the A2 group. Given that the D1 group excelled in growth performance and carcass characteristics and the D2 group excelled in carcass characteristics, we speculate that the 5% level of MOLP may have been achieved by adjusting the relative abundance ratios of Firmicutes, Bacteroidetes, Actinobacteria, and Tenericutes.

To more precisely analyze the effects of different MOLP levels on broiler chicken cecal microflora, the abundances of the genera of different groups were compared (Figure 5). In total, there were 8 dominant bacteria (relative abundance ≥1%) at the genus level. *Bacteroides* were the most abundant; this genus is adept at degrading and assembling polysaccharides, especially crude fiber [30], and has positive impacts on host immune systems [31]. The addition of MOLP consistently significantly increased the relative abundance of *Bacteroides* (*p* < 0.05), except at 3% on day 28 and 1% on day 56. Therefore, the higher crude fiber content in MOLP may facilitate the growth of *Bacteroides* and, in turn, improve the health status of broiler chickens. *Faecalibacterium* consists of anaerobic bacteria that are negatively correlated with inflammatory bowel disease and colorectal cancer [32]; they play an important role in producing energy that colonocytes use and anti-inflammatory products, such as butyrate and salicylic acid, that benefit intestinal health [33]. Interestingly, the same levels of addition at different stages resulted in completely opposite effects on the abundance of *Faecalibacterium*, indicating that higher MOLP levels inhibited the growth of *Faecalibacterium* growth in early-stage chickens. Alternatively, in late-stage chickens, with the maturation of the broiler chicken digestive system and cecal microenvironment, MOLP addition supported *Faecalibacterium* reproduction. The data on growth performance and carcass characteristics of early and late broilers support the above results, i.e., higher levels of MOLP inhibited the growth of fecal bacteria in early-stage chickens and, thus, triggered a decrease in production performance and carcass characteristics in the early higher MOLP group. However, in the late stage, due to the gradual improvement of the broiler digestive system, higher levels of MOLP had a beneficial effect on the production performance and carcass characteristics of broilers. For *Oscillospira*, the same trend was observed in the different stages; increasing MOLP levels resulted in a significantly reduced relative abundance (*p* < 0.05). *Oscillospira* has been reported to be highly positively associated with leanness [34]; therefore, inhibition of this bacteria could improve the growth performance of broilers. *Phascolarctobacterium* was found to produce short-chain fatty acids such as acetate and propionate, which, in turn, exerted beneficial effects on the host [35]. The additional effects of MOLP were similar to their influence on *Faecalibacterium*; *Phascolarctobacterium* growth was more sensitive to MOLP addition in early-stage broiler chickens, whereas late-stage chickens had greater adaptive ability. *Prevotella* has been reported to play an essential role in carbohydrate utilization and can convert carbohydrates into acetic acid, succinic acid, isobutyric acid, and lactic acid, which can be used directly by their host [36,37]. Our data showed that at day 28, MOLP levels greater than 3% significantly reduced the abundance of Prevotella (*p* < 0.05). Additionally, on day 56, the effect of addition was not consistent, and the abundance of Prevotella was significantly lower at MOLP levels of 3% and 5%, the reason for which further investigation is needed. From the levels of the genus, we can see that different levels of MOLP can affect broiler growth performance and chicken carcass characteristics by decreasing the relative abundance of harmful bacteria in the Oscillospira genera and increasing the relative abundance of beneficial bacteria in the Bacteroides genera in the early and late stages.

To analyze the similarity between each group, the cecal microbiota was clustered at the genus level by column on a heat map (Figure 6). On day 28, the groups A1 and B1 clustered together, indicating that the addition of 1% MOLP slightly changed the cecal microbiota compared to the control group, and the further addition of MOLP significantly altered the cecal microbiota. Consequently, early-stage broiler chickens were sensitive to the addition of MOLP. Alternatively, on day 56, broiler chickens had a greater adaptive ability in the presence of higher MOLP levels, which was reflected by the difference in cecal micro-organisms between the A2 and F2 groups, which clustered. To identify the specific bacterial taxa present under the addition of different levels of MOLP, the cecal microbiota in different groups were compared by using the LEfSe method [38]. As tested at different taxon levels (Figure 7), in early-stage chickens, with the MOLP level increased to 5%, the most dominant bacteria changed from *Oscillospira* to *Prevotella*; this may be attributed to the higher crude fiber content in the diet [39]. On day 56, it was found that *Oscillospira* was still one of the most dominant bacteria in the A1 and A2 groups. *Oscillospira* has previously been reported to be positively associated with leanness [34], which may not help facilitate the rapid growth of broiler chickens, and we deduced that the higher content of antinutritional factors in the rapeseed cake may be the cause of the high abundance of *Oscillospira* [40].

## 5. Conclusions

Our study showed different changes in the cecal microbial ecosystems of broiler chickens at different stages, and its changes are closely related to the growth performance and carcass characteristics of broilers. To date, this is the first report to analyze the effects of MOLP on broiler chicken cecal microbial diversity by using 16S rRNA gene sequencing. The data revealed that MOLP treatment significantly affected broiler chicken cecal microflora. Changes in microbiota usually have an impact on broiler growth performance, carcass characteristics, and other indicators, while our study also showed that late-stage chickens have a stronger ability to adapt to MOLP than early-stage chickens. This study showed the potential use of MOLP as a substitute for rapeseed cake and other protein source feeds. By comprehensively evaluating the effects of different levels of MOLP on the cecum microbiota, growth performance, and carcass characteristics of broilers, it was found that the optimal level of MOLP addition for broilers was 5% in both the early and late stages.

## Figures and Tables

**Table 1:** Ingredient and nutrient composition of the experimental diets.

Items	A1/A2	B1/B2	C1/C2	D1/D2	E1/E2	F1/F2
Ingredients (%)						
Corn	62.3	62.2	61.9	61.6	61.4	61.1
Moringa oleifera powder	0.000	1.00	3.00	5.00	7.00	9.00
Soybean meal	20.0	20.1	20.4	20.7	20.9	21.2
Rape seed cake	9.00	8.00	6.00	4.00	2.00	0.000
Wheat bran	5.00	5.00	5.00	5.00	5.00	5.00
Nacl	0.300	0.300	0.300	0.300	0.300	0.300
Limestone powder	1.30	1.30	1.30	1.30	1.30	1.30
Calcium hydrogen phosphate	1.70	1.70	1.70	1.70	1.70	1.70
Lys	0.100	0.100	0.100	0.100	0.100	0.100
DL-Met	0.100	0.100	0.100	0.100	0.100	0.100
Mineral premix	0.0800	0.0800	0.0800	0.0800	0.0800	0.0800
Vitamin premix	0.0200	0.0200	0.0200	0.0200	0.0200	0.0200
Choline chloride	0.100	0.100	0.100	0.100	0.100	0.100
Total	100	100	100	100	100	100
Nutrient composition						
ME (MJ/kg)	11.7	11.7	11.7	11.7	11.7	11.7
CP (%)	18.2	18.1	18.1	18.2	18.2	18.3
Lys (%)	0.800	0.790	0.790	0.780	0.790	0.790
Lys + Met (%)	0.730	0.750	0.750	0.740	0.740	0.750
Ca (%)	0.900	0.910	0.910	0.910	0.910	0.910
P (%)	0.450	0.440	0.450	0.450	0.450	0.450

ME: metabolizable energy, CP: crude protein, Lys: lysine, Met: methionine, Ca: calcium, and P: phosphorus. Vitamin and mineral premix were supplied as per kg of formula feed: vitamin A 12,000 IU, vitamin D3 2400 IU, vitamin E 18.35 mg, vitamin K3 2.65 mg, vitamin B1 2 mg, vitamin B2 6 mg, vitamin B12 0.025 mg, biotin 0.032 mg, folic acid 1.25 mg, pantothenic acid 14 mg, niacin 48 mg, copper 8 mg, zinc 76 mg, ferrum 80 mg, manganese 105 mg, selenium 0.2 mg, and iodine 0.35 mg.

**Table 2:** Effects of Moringa oleoresin substitute for different ratios of rapeseed cake on the growth performance of broiler chicken.

Time	Items	A1/A2	B1/B2	C1/C2	D1/D2	E1/E2	F1/F2
4 weeks	ADG (g/d)	18.7 ± 1.90 ^b^	22.8 ± 2.10 ^c^	23.2 ± 2.20 ^c^	22.9 ± 1.90 ^c^	16.3 ± 1.40 ^a^	18.4 ± 1.60 ^b^
	ADFI (g/d)	59.9 ± 4.10 ^a^	60.5 ± 4.30 ^a^	69.0 ± 4.80 ^c^	61.3 ± 5.10 ^a^	66.8 ± 5.30 ^b^	66.8 ± 5.50 ^b^
	TBG (kg)	11.0 ± 0.900 ^b^	13.6 ± 1.10 ^b^	13.5 ± 1.20 ^d^	11.7 ± 0.600 ^c^	9.90 ± 0.640 ^a^	10.9 ± 0.900 ^b^
	F/G	6.80 ± 0.400 ^c^	5.40 ± 0.500 ^a^	5.80 ± 0.500 ^a^	6.30 ± 0.500 ^b^	7.90 ± 0.600 ^d^	7.5 ± 0.600 ^d^
8 weeks	ADG (g/d)	12.2 ± 1.10	12.2 ± 1.60	12.2 ± 2.50	12.4 ± 1.00	12.2 ± 1.80	12.2 ± 1.80
	ADFI (g/d)	76.4 ± 6.80	76.4 ± 6.10	76.5 ± 6.50	76.4 ± 6.40	76.4 ± 5.90	76.3 ± 5.50
	TBG (kg)	4.50 ± 0.400	4.60 ± 0.400	4.60 ± 0.400	4.60 ± 0.500	4.50 ± 0.400	4.50 ± 0.400
	F/G	5.90 ± 0.500	5.90 ± 0.500	5.90 ± 0.500	5.90 ± 0.500	5.80 ± 0.400	5.90 ± 0.500

All of the data are expressed as the mean ± SEM. Statistical analysis of the data was performed by using the Duncan method to analyze the significance of differences between samples by using IBM SPSS software. Means with similar superscripts in the same column indicate that there are no significant differences (*p* > 0.05), and those with different superscripts in the same column indicate significant differences (*p* < 0.05).

**Table 3:** Effects of Moringa oleoresin leaf power on the carcass characteristics of broiler chicken fed a 4-week diet.

Items	A1/A2	B1/B2	C1/C2	D1/D2	E1/E2	F1/F2
MOLP addition ratio (%)	0.000	1.00	3.00	5.00	7.00	9.00
Pre-slaughter weight (g)	780 ± 88.9	797 ± 50.4	818 ± 103	826 ± 93.6	741 ± 93.1	744 ± 99.1
Slaughter weight (g)	709 ± 79.8 ^ab^	716 ± 46.9 ^ab^	747 ± 95.9 ^b^	755 ± 80.2 ^b^	649 ± 87.2 ^a^	678 ± 91.5 ^ab^
Half chamber weight (g)	633 ± 71.9 ^ab^	626 ± 40.1 ^ab^	672 ± 90.1 ^b^	677 ± 77.8 ^b^	577 ± 92.9 ^a^	605 ± 88.8 ^ab^
Full bore weight (g)	493 ± 57.9	502 ± 44.1	519 ± 68.7	520 ± 70.1	475 ± 122	466 ± 73.7
Slaughter rate (%)	91 ± 0.800 ^b^	89.9 ± 2.40 ^ab^	91.3 ± 1.50 ^b^	91.4 ± 1.20 ^b^	87.8 ± 6.40 ^a^	91.1 ± 1.80 ^b^
Semi-clear bore rate (%)	81.1 ± 2.20 ^bc^	78.6 ± 3.10 ^ab^	82.1 ± 1.50 ^c^	82.0 ± 1.40 ^c^	77.7 ± 5.40 ^a^	81.2 ± 2.20 ^bc^
Full net bore rate (%)	63.2 ± 1.60	62.9 ± 2.60	63.4 ± 1.30	62.9 ± 2.70	63.7 ± 2.20	62.5 ± 2.60

All of the data are expressed as the mean ± SEM. Statistical analysis of the data was performed by using the Duncan method to analyze the significance of differences between samples by using IBM SPSS software. Means with similar superscripts in the same column indicate that there are no significant differences (*p* > 0.05), and those with different superscripts in the same column indicate significant differences (*p* < 0.05).

**Table 4:** Effects of Moringa oleoresin leaf power on the carcass characteristics of broiler chicken fed an 8-week diet.

Items	A1/A2	B1/B2	C1/C2	D1/D2	E1/E2	F1/F2
MOLP addition ratio (%)	0.000	1.00	3.00	5.00	7.00	9.00
Pre-slaughter weight (g)	1095 ± 1.41 ^ab^	1001 ± 136 ^a^	1215 ± 82.2 ^bc^	1249 ± 113 ^c^	1051 ± 128 ^a^	1124 ± 146 ^ab^
Slaughter weight (g)	1029 ± 109 ^ab^	938 ± 107 ^a^	1011 ± 322 ^ab^	1152 ± 76.0 ^b^	987 ± 124 ^a^	1048 ± 146 ^ab^
Half chamber weight (g)	918 ± 107 ^ab^	840 ± 118 ^a^	982 ± 99.4 ^bc^	1037 ± 77.5 ^c^	910 ± 107 ^ab^	940 ± 125 ^abc^
Full bore weight (g)	731 ± 90.1 ^ab^	655 ± 113 ^a^	803 ± 73.5 ^bc^	829 ± 52.1 ^c^	706 ± 90.5 ^a^	728 ± 99.2 ^ab^
Slaughter rate (%)	93.9 ± 0.170	93.7 ± 2.00	83.6 ± 2.60	92.5 ± 4.00	93.8 ± 1.00	93.3 ± 1.00
Semi-clear bore rate (%)	83.8 ± 3.00 ^ab^	83.9 ± 2.00 ^ab^	80.7 ± 3.80 ^a^	83.5 ± 9.10 ^ab^	86.7 ± 2.40 ^b^	83.7 ± 2.40 ^ab^
Full net bore rate (%)	66.6 ± 2.40	65.2 ± 3.40	66.0 ± 1.70	66.8 ± 7.10	67.1 ± 2.60	64.8 ± 2.10

All of the data are expressed as the mean ± SEM. Statistical analysis of the data was performed by using the Duncan method to analyze the significance of differences between samples by using IBM SPSS software. Means with similar superscripts in the same column indicate that there are no significant differences (*p* > 0.05), and those with different superscripts in the same column indicate significant differences (*p* < 0.05).

**Table 5:** Estimation of the diversity of the 16S rRNA gene information of the cecum from the sequence analysis.

Sample ID	Reads	OTU	Chao	Ace	Shannon	Simpson
A1-1	46922	1153	1830	1663	7.45	0.990
A1-2	33904	1038	1594	1462	7.53	0.990
A1-3	59311	1202	1975	1762	7.40	0.990
A2-1	59971	1238	2045	1835	7.71	0.990
A2-2	49762	1122	1566	1614	7.59	0.990
A2-3	52948	1172	1721	1584	7.62	0.990
B1-1	55548	984	1694	1519	6.75	0.980
B1-2	31336	842	1310	1214	6.89	0.980
B1-3	59054	1058	1648	1579	7.12	0.980
B2-1	39108	1018	1457	1459	7.32	0.990
B2-2	59320	1158	1848	1727	7.24	0.990
B2-3	51137	1156	1959	1766	7.34	0.990
C1-1	59706	1108	1778	1648	7.27	0.990
C1-2	37951	1023	1580	1476	7.33	0.980
C1-3	40680	1030	1615	1504	7.33	0.990
C2-1	38061	990	1463	1377	7.34	0.990
C2-2	51964	1085	1610	1506	7.42	0.990
C2-3	46430	1160	1829	1693	7.52	0.990
D1-1	55874	1206	1949	1788	7.56	0.990
D1-2	58446	1254	2058	1885	7.59	0.990
D1-3	59689	1271	2099	2016	7.49	0.990
D2-1	36073	1063	1742	1655	7.41	0.990
D2-2	44216	1181	1850	1770	7.62	0.990
D2-3	53262	1151	1763	1659	7.46	0.990
E1-1	31428	935	1413	1396	7.20	0.980
E1-2	58666	1133	1765	1676	6.93	0.980
E1-3	38584	1046	1514	1475	7.44	0.990
E2-1	32957	954	1455	1349	7.09	0.980
E2-2	39017	956	1314	1313	6.96	0.980
E2-3	49353	1108	1764	1618	6.99	0.980
F1-1	40044	1037	1598	1521	7.41	0.990
F1-2	49833	1128	1759	1619	7.48	0.990
F1-3	59982	1215	2166	1862	7.44	0.990
F2-1	43058	1064	1530	1478	7.55	0.990
F2-2	40579	1091	1655	1587	7.59	0.990
F2-3	42801	1063	1723	1553	7.54	0.990

**Table 6:** The relative abundance of dominant bacteria from different groups on day 28.

	A1	B1	C1	D1	E1	F1
Phylum						
Actinobacteria	0.0460 ± 0.0060	0.0410 ± 0.0090	0.0520 ± 0.0050	0.0180 ± 0.0040	0.0240 ± 0.0060	0.0350 ± 0.0020
Bacteroidetes	0.295 ± 0.0310	0.528 ± 0.0550	0.433 ± 0.0590	0.315 ± 0.0300	0.363 ± 0.0650	0.419 ± 0.0480
Firmicutes	0.603 ± 0.0240	0.393 ± 0.0440	0.470 ± 0.0550	0.579 ± 0.0340	0.551 ± 0.0550	0.495 ± 0.0440
Tenericutes	0.0290 ± 0.0030	0.0140 ± 0.0040	0.0190 ± 0.0030	0.0520 ± 0.0030	0.0390 ± 0.0090	0.0270 ± 0.0050
Genus						
Bacteroides	0.100 ± 0.0130	0.162 ± 0.0300	0.100 ± 0.0100	0.152 ± 0.0190	0.191 ± 0.0330	0.139 ± 0.0160
Dorea	0.0190 ± 0.0030	0.0230 ± 0.0030	0.0160 ± 0.0020	0.0160 ± 0.0010	0.0210 ± 0.0020	0.0220 ± 0.0050
Faecalibacterium	0.0680 ± 0.0050	0.0520 ± 0.0040	0.0550 ± 0.0160	0.0590 ± 0.0100	0.0680 ± 0.0040	0.0610 ± 0.0160
Oscillospira	0.0610 ± 0.0020	0.0390 ± 0.0050	0.0460 ± 0.0030	0.0500 ± 0.0010	0.0530 ± 0.0040	0.0430 ± 0.0010
Parabacteroides	0.0190 ± 0.0020	0.0410 ± 0.0040	0.0320 ± 0.0030	0.0170 ± 0.0010	0.0130 ± 0.0030	0.0400 ± 0.0050
Phascolarctobacterium	0.0380 ± 0.0030	0.0290 ± 0.0020	0.0320 ± 0.0030	0.0190 ± 0.0020	0.0230 ± 0.0030	0.0210 ± 0.0030
Prevotella	0.0660 ± 0.0050	0.104 ± 0.0100	0.106 ± 0.0180	0.0360 ± 0.0050	0.0740 ± 0.0150	0.0460 ± 0.0050
Ruminococcus	0.0430 ± 0.0010	0.0240 ± 0.0040	0.0370 ± 0.0050	0.0420 ± 0.0040	0.0390 ± 0.0060	0.0330 ± 0.0030

**Table 7:** The relative abundance of dominant bacteria from different groups on day 56.

	A2	B2	C2	D2	E2	F2
**Phylum**						
Actinobacteria	0.0110 ± 0.0010	0.0240 ± 0.0020	0.0130 ± 0.0030	0.0130 ± 0.0010	0.0110 ± 0.0020	0.0150 ± 0.0010
Bacteroidetes	0.435 ± 0.0360	0.499 ± 0.0190	0.507 ± 0.0510	0.397 ± 0.0490	0.544 ± 0.0130	0.564 ± 0.0090
Firmicutes	0.476 ± 0.0360	0.417 ± 0.0240	0.399 ± 0.0500	0.525 ± 0.0490	0.391 ± 0.0130	0.364 ± 0.0070
Tenericutes	0.0410 ± 0.0070	0.0150 ± 0.0020	0.0290 ± 0.0040	0.0330 ± 0.0060	0.0200 ± 0.0010	0.0210 ± 0.0020
**Genus**						
Bacteroides	0.109 ± 0.0080	0.116 ± 0.0090	0.139 ± 0.0070	0.144 ± 0.0110	0.229 ± 0.0060	0.156 ± 0.0070
Dorea	0.0110 ± 0.0010	0.0100 ± 0.0010	0.0090 ± 0.0010	0.0090 ± 0.0010	0.0100 ± 0.0010	0.0090 ± 0.0010
Faecalibacterium	0.0260 ± 0.0040	0.0350 ± 0.0020	0.0380 ± 0.0050	0.0640 ± 0.0060	0.0360 ± 0.0030	0.0360 ± 0.0010
Oscillospira	0.0610 ± 0.0070	0.0520 ± 0.0040	0.0430 ± 0.0030	0.0520 ± 0.0070	0.0420 ± 0.0020	0.0360 ± 0.0020
Parabacteroides	0.0390 ± 0.0040	0.0420 ± 0.0010	0.0500 ± 0.0050	0.0440 ± 0.0070	0.0370 ± 0.0030	0.0450 ± 0.0020
Phascolarctobacterium	0.0280 ± 0.0020	0.0370 ± 0.0020	0.0260 ± 0.0040	0.0260 ± 0.0020	0.0250 ± 0.0010	0.0260 ± 0.0010
Prevotella	0.0720 ± 0.0110	0.100 ± 0.0100	0.0580 ± 0.0070	0.0530 ± 0.0070	0.103 ± 0.0010	0.0880 ± 0.0010
Ruminococcus	0.0340 ± 0.0020	0.0310 ± 0.0030	0.0300 ± 0.0050	0.0460 ± 0.0050	0.0300 ± 0.0020	0.0290 ± 0.0020

## Data Availability

All the data from this study are included in the article, and the corresponding authors can be contacted directly for further queries.
